# Cognitive and Neurophysiological Effects of Non-invasive Brain Stimulation in Stroke Patients after Motor Rehabilitation

**DOI:** 10.3389/fnbeh.2016.00135

**Published:** 2016-06-24

**Authors:** Federico D'Agata, Elena Peila, Alessandro Cicerale, Marcella M. Caglio, Paola Caroppo, Sergio Vighetti, Alessandro Piedimonte, Alice Minuto, Marcello Campagnoli, Adriana Salatino, Maria T. Molo, Paolo Mortara, Lorenzo Pinessi, Giuseppe Massazza

**Affiliations:** ^1^Department of Neuroscience, University of TurinTurin, Italy; ^2^UO Neurology V - Neuropathology, Fondazione IRCCS Istituto Neurologico Carlo BestaMilano, Italy; ^3^Physical Medicine and Rehabilitation, University of TurinTurin, Italy; ^4^Molo ONLUSTurin, Italy

**Keywords:** non-invasive brain stimulation, transcranial magnetic stimulation, transcranial direct current stimulation, mirror-box therapy, stroke rehabilitation

## Abstract

The primary aim of this study was to evaluate and compare the effectiveness of two specific Non-Invasive Brain Stimulation (NIBS) paradigms, the repetitive Transcranial Magnetic Stimulation (rTMS), and transcranial Direct Current Stimulation (tDCS), in the upper limb rehabilitation of patients with stroke. Short and long term outcomes (after 3 and 6 months, respectively) were evaluated. We measured, at multiple time points, the manual dexterity using a validated clinical scale (ARAT), electroencephalography auditory event related potentials, and neuropsychological performances in patients with chronic stroke of middle severity. Thirty four patients were enrolled and randomized. The intervention group was treated with a NIBS protocol longer than usual, applying a second cycle of stimulation, after a washout period, using different techniques in the two cycles (rTMS/tDCS). We compared the results with a control group treated with sham stimulation. We split the data analysis into three studies. In this first study we examined if a cumulative effect was clinically visible. In the second study we compared the effects of the two techniques. In the third study we explored if patients with minor cognitive impairment have most benefit from the treatment and if cognitive and motor outcomes were correlated. We found that the impairment in some cognitive domains cannot be considered an exclusion criterion for rehabilitation with NIBS. ERP improved, related to cognitive and attentional processes after stimulation on the motor cortex, but transitorily. This effect could be linked to the restoration of hemispheric balance or by the effects of distant connections. In our study the effects of the two NIBS were comparable, with some advantages using tDCS vs. rTMS in stroke rehabilitation. Finally we found that more than one cycle (2–4 weeks), spaced out by washout periods, should be used, only in responder patients, to obtain clinical relevant results.

## Introduction

Motor and cognitive impairment are frequent aftermaths of brain damage after a stroke. Many authors reports cognitive deficits in 12–56% of stroke patients and reduced performances in several cognitive domains in 32% (Ebrahim et al., 1985; Tatemichi et al., 1994; Patel et al., 2002). Moreover, dysfunctions in the use of upper limb and in functional walking are among the more common consequences for many stroke survivors. Of note, only 5% of adult stroke survivors regain full function of the upper limb and 20% do not recover any functional use.

The severity of cognitive impairment negatively correlates with motor and functional recovery achieved in stroke patients after rehabilitation. Indeed, a cognitive assessment should be used to select patients that could have the best benefits from rehabilitation (Patel et al., 2002; Mehta et al., 2003; Saxena et al., 2007; Rabadi et al., 2008).

Event Related Potentials (ERP) are a reproducible electrophysiological response to an external stimulus (visual or auditory), representing the brain activity associated with various cognitive processes such as selective attention, memory, or decision making. Interestingly, ERP can be valuable in the diagnosis of cognitive impairment and are able to track the cognitive changes during the follow-up in stroke patients (Trinka et al., 2000; Alonso-Prieto et al., 2002; Yamagata et al., 2004; Stahlhut et al., 2014).

Recently, Non-Invasive Brain Stimulation (NIBS) techniques have been proposed as support of standard cognitive and motor rehabilitation. The application of NIBS in stroke rehabilitation arises from the observation that cortical excitability can be modulated after electrical or magnetic brain stimulation. It can be reduced or enhanced (Miniussi et al., 2008; Sandrini and Cohen, 2013) depending on many factors (stimulation parameters, type of stimulation technique, timing of the stimulation, brain target region, and state of mind).

The physiological mechanisms underlying brain stimulation effects are still partially unknown, but several evidences explain these effects with Long Term Potentiation (LTP) and Long Term Depression (LTD) like mechanisms (Thickbroom, 2007; Fritsch et al., 2010; Bliss and Cooke, 2011).

Repetitive Transcranial Magnetic Stimulation (rTMS) and transcranial Direct Current Stimulation (tDCS) are the most used NIBS techniques in rehabilitation (Hummel et al., 2005; Miniussi et al., 2008; Bolognini et al., 2009). Both can induce long lasting effect on cortical plasticity (30–90 min). Modification of cortical activity may improve the subject's ability to relearn or acquire new strategies for carrying out motor or behavioral task, by facilitating perilesional activity or by suppressing maladaptive interfering activity from other brain areas (Miniussi et al., 2008). Even if most of the effects are transient, NIBS during or before a learning process may yield the behavioral improvements more robust and stable (Rossi and Rossini, 2004; Pascual-Leone, 2006). Indeed, during motor learning not only the fast (intra-sessions) and slow (inter-sessions) learning during training are relevant, but also the memory consolidation and the savings (Wessel et al., 2015). Plasticity induced by NIBS could thus have important effects not only in the online phase of motor rehabilitation, but also in the offline phases.

A growing number of studies indicates that NIBS could be useful in chronic stroke rehabilitation (Hummel and Cohen, 2006; Sandrini and Cohen, 2013; Liew et al., 2014; Wessel et al., 2015), but no one compared directly the two techniques or explored the link between cognitive and motor improvement. TMS is able to directly induce action potentials in the axons while the currents used in tDCS (1–2 mA) cannot. The first technique is, therefore, best suited to be used offline, while the second can be used online in conjunction with other rehabilitation techniques or tasks (Wessel et al., 2015). Simis et al. (2013) compared rTMS and tDCS in healthy subjects, observing that both techniques induced similar motor gains. The comparison of brain plasticity induced by NIBS in pathologic subjects could thus extend significantly the Simis' results.

In this paper, the primary aim was to evaluate and compare the motor and cognitive changes induced by rTMS and tDCS in the upper limb rehabilitation in patients with stroke, both in short and in long term outcome. Secondarily we searched for a possible link between motor and cognitive measures.

We chose the most effective paradigm of rTMS in chronic stroke according to meta-analyses and consensus papers (Lefaucheur et al., 2014), a low-frequency protocol applied onto the controlesional motor cortex (M1). For tDCS, in the absence of a gold standard, we chose a paradigm with a dual sites montage validated in non-inferiority trials (Schlaug et al., 2008; Lüdemann-Podubecká et al., 2014). The tDCS was performed in conjunction with a cognitive training focused on the brain representation of the hands, the mirror-box therapy (MT), to direct the neuromodulation effect as wished. Our aim was to create a paradigm easy to apply in a clinical setting.

To compare the NIBS techniques in the same patients we created a treatment longer than usual applying a second cycle of stimulation, after a washout period, using different techniques in the two cycles (rTMS/tDCS).

A randomized clinical trial divided into three studies was designed to explore the following issues:

A longer NIBS stimulation could be beneficial in stroke rehabilitation?
What are the differences between rTMS and tDCS in stroke rehabilitation?
NIBS motor stimulation effects can modulate or be modulated by patients' cognitive status?

In the first study we evaluated if a cumulative effect, mediated by an offline improvement (consolidation or savings), was clinically detectable. We also evaluated the differences between a first priming cycle with rTMS followed by tDCS and first priming with tDCS followed by rTMS.

In the second study we compared the effects of the two techniques to test if brain plasticity effects could depend on the type of NIBS. In the third study, we searched for a possible link between motor and cognition changes, evaluating if cognitive measures changed in patients with motor improvement differently from the patients without motor improvement.

## Materials and methods

### Patients

Thirty four consecutive patients (see Table 1 for demographic and clinical data), with chronic ischemic or hemorrhagic stroke (>6 months from the accident), aged between 18 and 70 years, attending the Physical Medicine and Rehabilitation department at AOU Città della Salute e della Scienza—Presidio Molinette Hospital in Torino, Italy, were enrolled in the study. Exclusion criteria were: global cognitive impairment (Mini Mental State Examination < 25), severe functional disability (Barthel Index < 45), severe psychiatric disorders, degenerative neurological disorders, epilepsy, and severe medical conditions. Patients with implanted drug infusion systems, spinal/brain-stimulators, or endovascular coils were excluded. In accordance with institutional guidelines and the Declaration of Helsinki, the local ethics committee gave approval to this study and all the involved participants signed informed written consent. The study was registered on ClinicalTrials.gov, identifier: NCT02525393. We selected a sample size similar to the numbers usually used in literature (about 31 subjects for rTMS and 30 for tDCS, means estimated from Pollock et al., 2014).

**Table 1:** **Clinical and demographic data**.

**Variable**	**Intervention**	**Sham**	p^*^
N	24	10	–
Age [years]	57 (12)	65 (12)	0.079
Gender M/F (%)	67/33%	70/30%	0.999
Education [years]	10 (4)	10 (4)	0.869
Affected hemisphere R/L (%)	50/50%	40/60%	0.715
Etiopathogenesis (%)			0.999
Ischemic	71%	70%	
Hemorrhagic	29%	30%	
Previous stroke events (%)	21%	30%	0.666
Lesion localization (%)			0.735
Cortical	25%	10%	
Subcortical	62%	80%	
Both	13%	10%	
Time from stroke [mos]	41 (39)	37 (32)	0.797
Smoke (%)	25%	10%	0.644
Hypertension (%)	72%	70%	0.999
Diabetes (%)	8%	30%	0.138
Dyslipidemia (%)	38%	40%	0.999
Stroke familiar history (%)	21%	30%	0.666
**THERAPY**			
Antidepressant	71%	40%	0.130
Antihypertensive	58%	60%	0.999
Antiplatelet	79%	70%	0.666
**NIBS SEQUENCE**			
TMS-tDCS	8	–	–
tDCS-TMS	16		
SIDE EFFECTS IN 10 SESSIONS^**^			
TMS	4		–
tDCS	6	3	
Drop outs (%)	24%	0%	–

*Mean and (standard deviation) or frequency*,

* *p, probability for two sample independent t-test or Kruscal–Wallis or Fisher's exact test*.

** *tiredness or headache for sham, tDCS and TMS, transient hearing loss for TMS*.

### Experimental design

**Figure 1:**
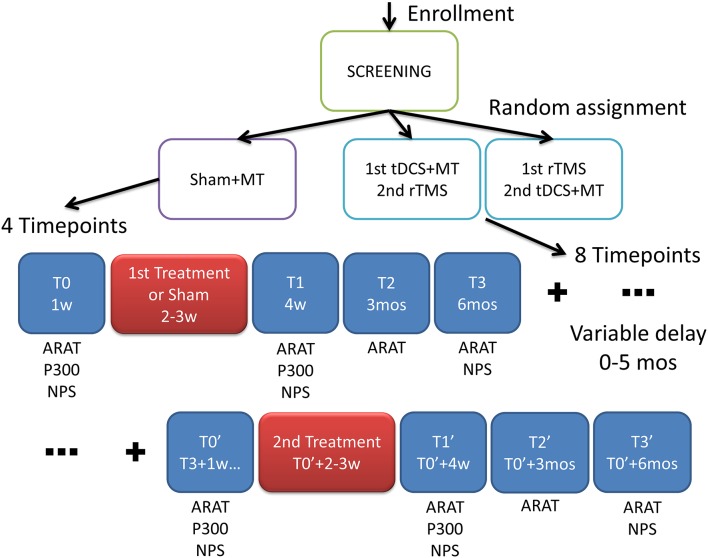
**Experimental design**. After screening the patients were randomized into three groups with different interventions: MT, Mirror Therapy; tDCS, transcranial Direct Current Stimulation; rTMS, repetitive Transranial Magnetic Stimulation. In the scheme the outcome measures: ARAT, Action Research Arm Test; P300, cognitive auditory evoked response potentials; NPS, neuropsychological test where assessed in multiple time frames; w, week; mos, months.

The trial was randomized double blind (Subject, Caregiver, Outcomes Assessor), interventional, with a factorial design (see Figure 1). Patients were randomly assigned to 3 arms:

The first intervention group (rTMS+tDCS, *N* = 16) received 10 daily sessions of rTMS for 2 weeks and after a washout period (at least 6 months) 10 daily sessions of dual-tDCS + MT for 2 weeks.

The second intervention group (tDCS+rTMS, *N* = 8) received dual-tDCS + MT and then rTMS, after washout.

A control group (*N* = 10) received 10 daily sessions of sham-tDCS + MT for 2 weeks.

The primary outcome measure was the Action Research Arm Test (ARAT) a quantitative upper extremity function test. The endpoint for a successful intervention was set, considering the Minimal Clinically Important Difference (MCID/MCD), in a range between 3.5 and 5.7 points (Van der Lee et al., 2001; Lin et al., 2009).

The secondary outcome measures were cognitive functions evaluated by electroencephalography (EEG) auditory evoked response potentials (ERP) and neuropsychological paper and pencil tests (NPS).

Time frames for the outcome measurements and interventions administration are shown in Figure 1.

Interventions were administered at weeks 2–3 and at around weeks 26–27 (6 months and 2–3 weeks). At baseline (T0) ARAT, ERP and NPS were assessed, at T1 (4 weeks) ARAT, ERP and NPS, at T2 (3 months) ARAT, at T3 (6 months) ARAT and NPS, at T0′ (6–11 months and 1 week) ARAT, ERP and NPS, at T1′ (7–12 months) ARAT, ERP and NPS, at T2′ (9–14 months) ARAT, at T3′ (12–17 months) ARAT and NPS.

The study was realized in a clinical setting so we decide to apply both stimulations (rTMS, tDCS) in the same patients to compare the safety and effectiveness of the techniques intra-patient, overcoming the problem of the sample's heterogeneity.

Demographic or clinical variables did not significantly differ between the patients that received the interventions and the control group (see Table 1).

### Action research arm test

The ARAT is a quantitative scale with 19 tasks graded from 0 to 3 (0 = cannot execute the task, 3 = can perform normally). It has four subscales: grasp (6 tasks), grip (4 tasks), pinch (6 tasks), and gross movements (3 tasks). In the first three subscales the tasks consist in grasping, moving and releasing different objects (e.g., wood block, glass, tube, ball), the last scale consists of three large movements (i.e., place hand behind the head, place hand on the top of the head, and move hand to mouth). Summing the scores, ARAT ranges from 0 to 57; higher scores indicate better upper extremity functionality. It is a reliable, valid, and standardized functional assessment tool with good to excellent psychometric characteristics (Van der Lee et al., 2001; Connell and Tyson, 2012; Pandian and Arya, 2014).

### Event related potentials

Electroencephalography data were recorded using a 20 channel EEG Galileo Star System (Esaote Biomedica, Verona). The patient was placed in a comfortable seated position, controlled by a technician in order to prevent drowsiness and limb movements. Nineteen standard scalp electrodes were applied to the scalp in accordance with the 10–20 International System (Fz, F1, F2, F3, F4, F7, F8, C3, C4, Cz, P3, P4, Pz, T3, T4, T5, T6, O1, O2). Impedance was < 2 kΩ in each active lead before the starting of the recording, and the reference was obtained by averaging the channels. Data were collected for each subject and digitized at a sampling rate of 256 Hz, with a band pass filter of 0.1–70 Hz and a notch filter to remove the main electrical noise in each channel. EEG was recorded for 5 min periods at rest (baseline). Electrooculography was simultaneously recorded with two electrodes placed near the left eye to detect and reject ocular movement artifacts from EEG data offline. ERP was recorded using the auditory oddball paradigm. The subjects were asked to react by counting target stimuli appearing rarely (r) amongst a series of more common stimuli (f) administered bilaterally by stereophonic earphones at 100 dB. Rare stimuli consisted in a 1500 Hz pure tone and frequent stimuli consisted in a 1000 Hz pure tone, presented in random order and with a mean r/f ratio of 1 r every 6 f. The recorded signal was cut appropriately (the time window started 200 ms before and ended 1100 ms after the stimulus) and ERP were averaged separately for the rare and frequent stimulus (using a mean of 20 stimuli), and the latencies of endogenous N200 and P300 components were evaluated according to the international recommendation (Goodin et al., 1994).

### Neuropsychological assessment

The patients were evaluated by a standardized neuropsychological assessment consisting of a battery of cognitive tests (described below) involving the following domains: verbal short-term memory, visuospatial learning, working memory, verbal learning, attention and frontal executive functions, and general cognitive impairment. Parallel and equivalent forms were used for all tests; we used standardized tests with normative values for the Italian population (Spinnler and Tognoni, 1987).

#### Mini mental state examination (MMSE)

This test is the most widely used, single measure of global cognitive functioning. It is a screening tool and it is utilized in evaluating mental state in research and clinical practice, testing global cognitive impairment.

#### Forward and backward digit span

The participant has to remember lists of increasing length of single-digit numbers and recalls them in the right and in the opposite order. Performance is defined by the longest sequence at which participants correctly recall at least two out of three sequences. This test is a measure of mental tracking as well as brief storage and mental manipulation.

#### Attentional matrices

It is used to evaluate attentional functions, in particular selective and sustained attention. Three matrices of numbers are administered with the instruction to cross out as fast as possible target numbers of either one, two, or three digits. The purpose of this test is to assess the subjects' ability to detect visual targets among distractors.

#### Short story test

This test assesses verbal memory function; the experimenter reads a short story only once and then the examinees should recall as much as they can immediately upon finishing. After 10 min have passed the examinee should repeat the recall.

#### Copy of figure

This test is used to assess visuospatial and visuoconstructive skills, visual memory, and executive functioning. The examinees are asked to reproduce a drawing, first by copying it freehand, and then drawing from memory.

#### Visual search and cancellation tasks

The number of items omitted is an indication of vigilance and the proportion of items omitted in each quadrant of the test page can suggest the presence of a possible unilateral spatial neglect.

#### Nelson MCST

This test is an abbreviated and Modified version of the Wisconsin Card Sorting Test; it assesses many aspects of executive functioning including mental flexibility and concept formation.

#### Hamilton depression rating scale (HDRS)

It is the most widely used clinician-administered depression assessment scale. A limitation of the HDRS is that atypical symptoms of depression are not assessed (Hamilton, 1980).

### Stimulation protocols

The main target of the stimulation protocols was to normalize the inter-hemispheric inhibition that is generally altered in stroke patients (Wessel et al., 2015). It has been demonstrated that anodal tDCS/high frequency rTMS applied to ipsilesional M1 and cathodal tDCS/low frequency applied to contralesional M1 can improved motor functions of the affected upper limb in chronic stroke patients (Sandrini and Cohen, 2013; Liew et al., 2014; Wessel et al., 2015).

Inhibitory low frequency rTMS (1 Hz) was administrated using a PowerMAG 100 device (MAG&More, München), at 80% resting motor threshold, for 15 min (900 stimuli) over the intact M1 with an eight-shaped coil. The coil was placed on M1, aiming for cortical area coding for hands' movement.

Single pulse TMS was used to determine bilateral M1 hot spots for the first dorsal interosseus (FDI) muscles defined as the place onto the scalps where the motor evoked potential (MEP) was maximum. MEP where obtained with the 120% of the minimum intensity required eliciting electromyography activity of at least 50 μV peak-to-peak amplitude in ≥50% of pursued trials (≥3/6) with the muscle at rest.

We registered surface electromyography with the Neurowerk EMG (Sigma Medizin–Technik, Gelenau/Erzgebirge) moving the coil in a grid of 0.5-cm steps medial, lateral, posterior, and anterior until the point of the maximum MEP was ascertained. The procedure was repeated iteratively until the hot spot was identified. The distances between C3/C4 and TMS hotspots were noted bilaterally for all patients. Cortical targets were identified using SofTaxic neuronavigation system (EMS, Bologna) along sessions.

tDCS was administrated using a HDC Stim device (Newronica, Milan), via two 5 × 5 cm^2^ pads (one anode and one cathode) soaked with saline solution. The tDCS was applied with the cathode onto the controlesional M1 and the anode onto the perilesional M1, the stimulation was online together with mirror-box therapy (MT). The anode (stimulating activity) was placed on the damaged hemisphere in the area corresponding to C3 or C4 position in the 10–20 systems, while the cathode (inhibitory activity) was placed in the analog position on the opposite hemisphere in a dual-tDCS design. The intensity of the stimulation was set at 1.5 mA and the duration of the tDCS was set at 20 min.

In our sample the mean distance between hotspots and C3/C4 was 2.1 ± 1.7 cm for the unaffected hemisphere and 2.4 ± 1.5 cm for the lesioned hemisphere; around 67% of the hotspots were inside the areas covered by pads.

### Mirror box therapy

MT consisted in the optical illusion of bimanual movements created by a box with a mirror in the middle, it has been ideated by Ramachandran et al. (1995) to treat phantom limb pain and has also been widely used as a rehabilitation tool after stroke (Dohle et al., 2009).

The box consisted in a wooden enclosure separated in two sections by a mirror. The patients had to insert his/her hand through the holes situated on the side of the box and could watch the normal hand while performing the requested gestures. The sensation of the movement of the plegic hand was generated when the patients looked at the reflection of the normal hand during the exercises. During the tDCS application the patient had to execute 3 series of 25 repetitions of 6 different movements (e.g., the hitcher gesture). The exercises were changed completely between the first and the second week of intervention.

Only tDCS stimulation was paired with MT to enhance its specificity to reach the level of focal rTMS targeting the hand area. In fact, one way to gain specificity is to have a precise simulation of the delivered power on a small anatomical area of interest (e.g., HD-tDCS; Bikson et al., 2013), but a simpler way is the activity-selectivity technique. tDCS will preferentially modulate specific forms of ongoing activity, so we paired it with an online task focused on hand movement to boost specifically ongoing plasticity activated by the task (Bikson et al., 2013).

### Statistical analysis

We adopted parametric statistics (*t*-test, ANOVA) when needed, otherwise we adopted non parametric tests (χ^2^ or Fisher's Exact test, Kruskal–Wallis's H, Friedman's test). For *post-hoc* comparisons we used Tukey HSD or Simes corrections (respectively for parametric and non-parametric tests).

The differences between groups at baseline for clinical, demographic, or outcome measures were tested.

As we tested multiple hypotheses with three different studies we also controlled if the significative results (*p* < 0.05) are still significant after a Bonferroni correction (*p* < 0.017) for multiple comparisons.

### Study 1—clinically efficacy and safety of two cycles of NIBS

To compare the efficacy of the treatment vs. sham for the ARAT outcome we used the repeated measures ANOVA (4 time frames: baseline, after interventions, short, and long follow-up) and one between factor (3 levels: tDCS+MT+rTMS, rTMS+tDCS+MT, sham+MT). In addition, we compared, with the same model, the sham group and the subgroup of responders, defined as patients that get an improvement in ARAT score after the first stimulation, to look if responders reached the MCID/MCD. Finally, we used a repeated measures ANOVA (8 time frames: baseline, after one cycle of NIBS, short and long follow-up, after pause, after second cycle of NIBS, second short and long follow-up) and one between factor (priming stimulation tDCS or rTMS) to look if the second cycle could be useful and if the order of priming stimulation was relevant on outcome.

Similar model were used for ERP and NPS with appropriate repetitions over time frames.

To compare ERP we used the repeated measures ANOVA (2 time frames: baseline and after interventions and 10 electrodes: F3, F4, F7, F8, C3, C4, P3, P4, T3, T4) and one between factor (3 levels: tDCS+MT+rTMS, rTMS+tDCS+MT, sham+MT). In addition, we used the repeated measures ANOVA (4 time frames: baseline, after one cycle of NIBS, after pause, after second cycle of NIBS) and one between factor (priming stimulation tDCS or rTMS).

To compare NPS we used the repeated measures ANOVA (3 time frames: baseline, after interventions and 6 months follow-up) and one between factor (3 levels: tDCS+MT+rTMS, rTMS+tDCS+MT, sham+MT). In addition, we used the repeated measures ANOVA (6 time frames: baseline, after one cycle of NIBS, long follow-up after pause, after second cycle of NIBS, second long-follow-up) and one between factor (priming stimulation tDCS or rTMS).

### Study 2—comparison of tDCS and rTMS clinical efficacy

To compare tDCS and rTMS induced changes for ARAT, ERP, and NPS intra-patients in the real stimulations groups we used a repeated measures ANOVA with time (4, 2, or 3 levels for ARAT, ERP, and NPS, respectively) and type of NIBS (two levels) as factors.

We also tested if rTMS and tDCS had a similar level of specificity looking at the profiles of ARAT subscales that, in our hypothesis, should be improved in a similar way by the two stimulations.

### Study 3—cognitive differences in patients that responded to motor rehabilitation

We also looked if responders had some differences in the cognitive measures (ERP and NPS) at baseline or after one or after two cycles of stimulations using repeated measure ANOVA with time (3 levels, within) and responders vs. no-responders (between) as factors.

## Results

### Patients

ARAT did not differ at baseline (*p* = 0.212).

At baseline, there were significant differences for the N200 and P300 in the study group. Indeed the sham group had shorter latencies compared to other groups (grand mean: N200 *F* = 9.1, *p* = 0.001 *post-hoc* sham < rTMS+tDCS, tDCS+rTMS; P300 *F* = 10.1, *p* < 0.001 *post-hoc* sham < rTMS+tDCS, tDCS+rTMS). Considering the P300 grand mean, latencies were over a normative cut off, determined on the basis of published data (Dinteren et al., 2014), without significant difference among groups (sham = 10%, rTMS+tDCS = 33%, tDCS+rTMS = 33%, Fisher = 2.0, *p* = 0.498).

Neuropsychological score did not differ between groups (Table 2). The total sample did not have general severe cognitive impairment (MMSE % patients Under normative Cut Off, UCO = 0%), but presented several focal deficits in many domains, especially in speed processing, attention and visuospatial skills (UCO 26–51%, Table 2).

**Table 2:** **Neuropsychological scores**.

**Test**	**Sham**	**rTMS+tDCS**	**tDCS+rTMS**	p^*^	UCO^**^
MMSE	28.1	26.1	27.5	0.572	0
Digit span (Forward)	5.70	4.63	5.00	0.074	15%
Digit span (Backwards)	4.00	3.22	3.87	0.511	–
Attentional matrices	42.2	41.9	40.0	0.845	24%
Short story	13.5	11.7	13.5	0.713	9%
Copy of figure delayed recall	0.65	0.61	0.48	0.184	21%
Copy of figure immediate recall	0.70	0.71	0.67	0.093	6%
Cancellation task (Total omissions)	1.40	1.74	3.39	0.221	26%
Cancellation task (Time)	138	136	144	0.527	51%
Nelson MCST (Categories)	4.3	4.4	5.0	0.317	12%
Nelson MCST (Perseverations)	4.8	4.7	3.3	0.350	21%
Hamilton rating depression scale	3.7	4.3	2.7	0.440	0

* p, probability for Friedman test;

** *UCO, percent of patients Under Cut Off in the sample as a whole*.

### Study 1—clinically efficacy and safety of two cycles of NIBS

We did not find any significant effect on ARAT scores for time (*F* = 0.7, *p* = 0.523), group (*F* = 1.1, *p* = 0.355) or their interaction (*F* = 2.2, *p* = 0.153).

**Figure 2:**
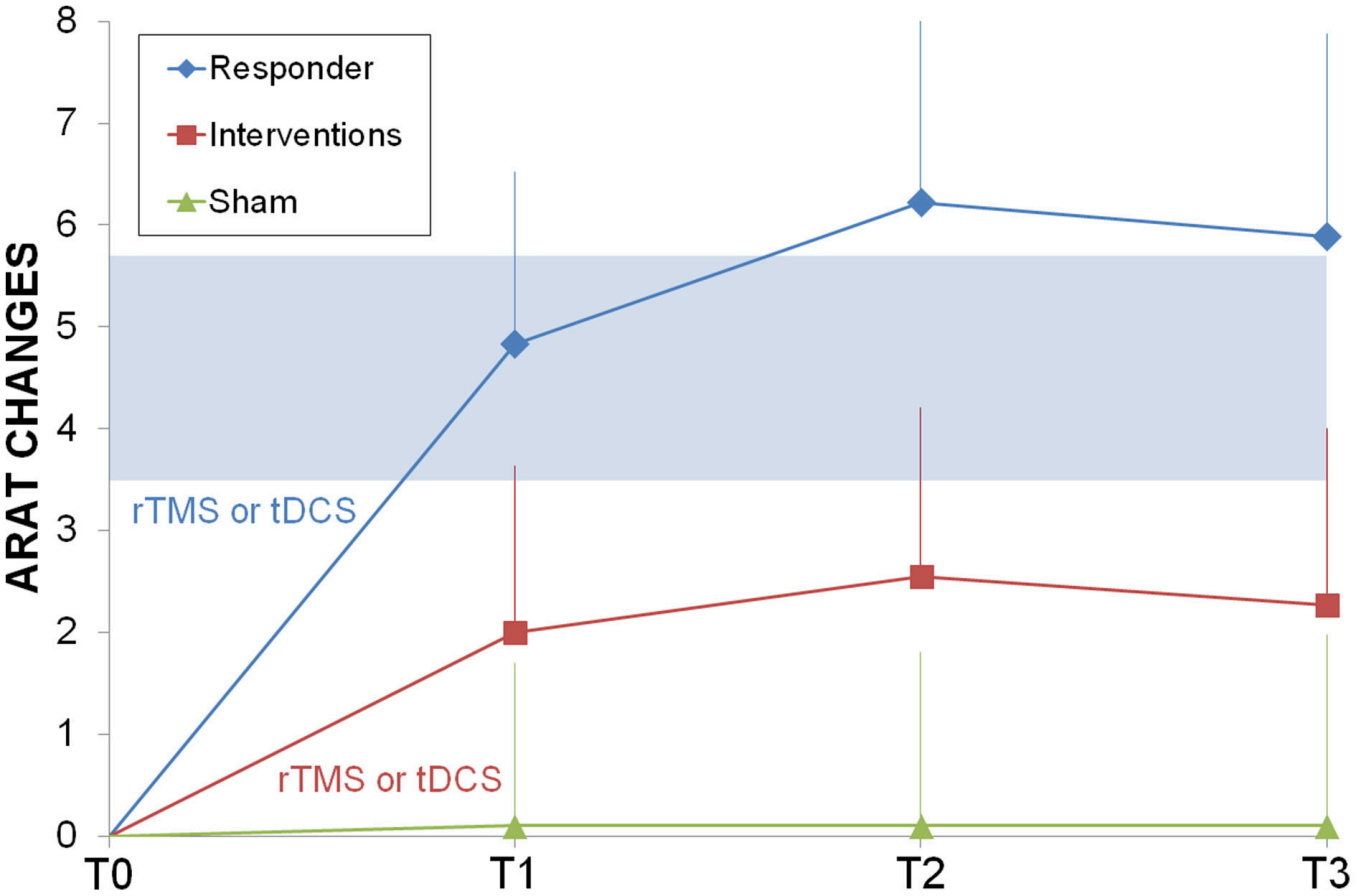
**Longitudinal psysiatric evaluation**. ARAT changes from baseline were shown for sham control group (light green triangle), interventions group (rTMS or tDCS, red squares) and responder subgroup (ARAT T1> ARAT T0, blue diamonds). In light blue the range of Minimally Important Clinical Difference. Abbreviations as in Figure 1.

The responders subgroup included the 44% of the treated patients, while only 20% of sham patients improved their ARAT score; all responders improved also after the second intervention and the gains were stable in the 75% of cases after 6 months. Most (90%) non-responders did not improve after the second intervention. When comparing responders vs. sham (Figure 2), ARAT showed a significant effects for time (*F* = 4.1, *p* = 0.012, η^2^ = 0.2) and time by group (*F* = 3.9, *p* = 0.015, η^2^ = 0.2), but not for group (*F* = 0.4, *p* = 0.531). It is evident that the effect of time and the interaction is due to the responders that reached the MCID/MCD range (see means in Figure 2). Results survived Bonferroni correction (*p* < 0.016).

**Figure 3:**
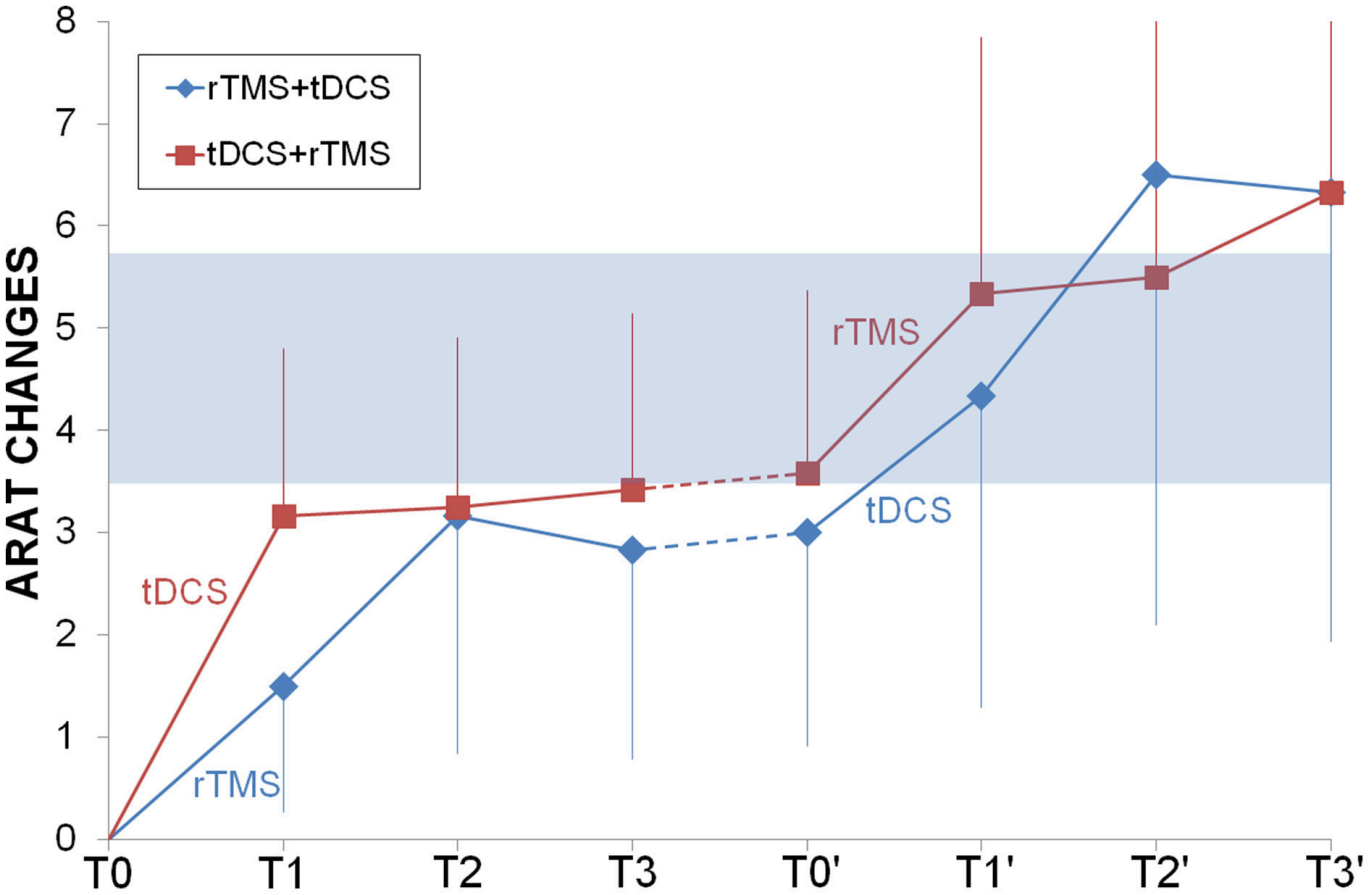
**Longitudinal comparison between rTMS and tDCS**. ARAT changes from baseline were shown for rTMS+tDCS (blue diamonds) and tDCS+rTMS (red squares) groups. Dotted lines indicated washout. In light blue the range of Minimally Important Clinical Difference. Abbreviations as in Figure 1.

ARAT had a very similar progression for both groups and the two sequential protocols (Figure 3), reaching the MCID/MCD range only after the second intervention. Only time had a significant effect (*F* = 3.54, *p* = 0.002) but not priming stimulation (*F* = 0.35, *p* = 0.565) or interaction (*F* = 0.13, *p* = 0.99), the *post-hoc* analysis showed that there were higher values at the end of the second cycle of NIBS (second long follow-up = second short follow-up = second cycle of NIBS > baseline, after one cycle of NIBS, short and long follow-up and after pause).

Time had a significant effect on both N200 (*F* = 41.9, *p* < 0.001, η^2^ = 0.2) and P300 (*F* = 115.1, *p* < 0.001, η^2^ = 0.4) latencies. The electrode was not significant for both N200 (*F* = 0.6, *p* = 0.830) and P300 (*F* = 0.4, *p* = 0.922). The intervention was significant on both N200 (*F* = 16.2, *p* < 0.001, η^2^ = 0.1) and P300 (*F* = 8.6, *p* < 0.001, η^2^ = 0.1). A significant interaction term time by intervention was found for N200 (*F* = 5.1 *p* = 0.007, η^2^ = 0.1) and P300 (*F* = 34.3, *p* < 0.001, η^2^ = 0.3). Results survived Bonferroni correction (*p* < 0.016). Other interactions terms were all not significant for N200 and P300.

In the *post-hoc* analyses the N200 and P300 latencies in the second time point were significantly shorter, sham group had shorter latencies at baseline, and interaction depends by a greater lowering in the rTMS+tDCS (N200 = 22 ms, P300 = 32 ms) and tDCS+rTMS (N200 = 21 ms, P300 = 36 ms) groups compared to sham (N200 = 6 ms, P300 = 1 ms).

Time was only significant for the copy of figure with immediate recall (*F* = 5.9, *p* = 0.006, η^2^ = 0.2), as was time by intervention interaction (*F* = 4.6, *p* = 0.004, η^2^ = 0.3), but not for intervention (*F* = 0.6, *p* = 0.568). In the *post-hoc* analyses rTMS+tDCS and tDCS+rTMS groups had a similar stable improvement that the sham group did not show.

For both ERP and NPS the sequence of priming was not significant.

### Study 2—comparison of tDCS and rTMS clinical efficacy

The change in ARAT score did not differ between tDCS or rTMS neither in the total sample nor in the responders subgroup. Also, the ARAT profiles were similar for rTMS and tDCS. Respectively, the scores were: gross movements 0.8 vs. 0.7, grasp 1.6 vs. 2.3, grip 0.4 vs. 0.8, pinch 0.9 vs. 0.7, showing the same qualitative improvements.

**Figure 4:**
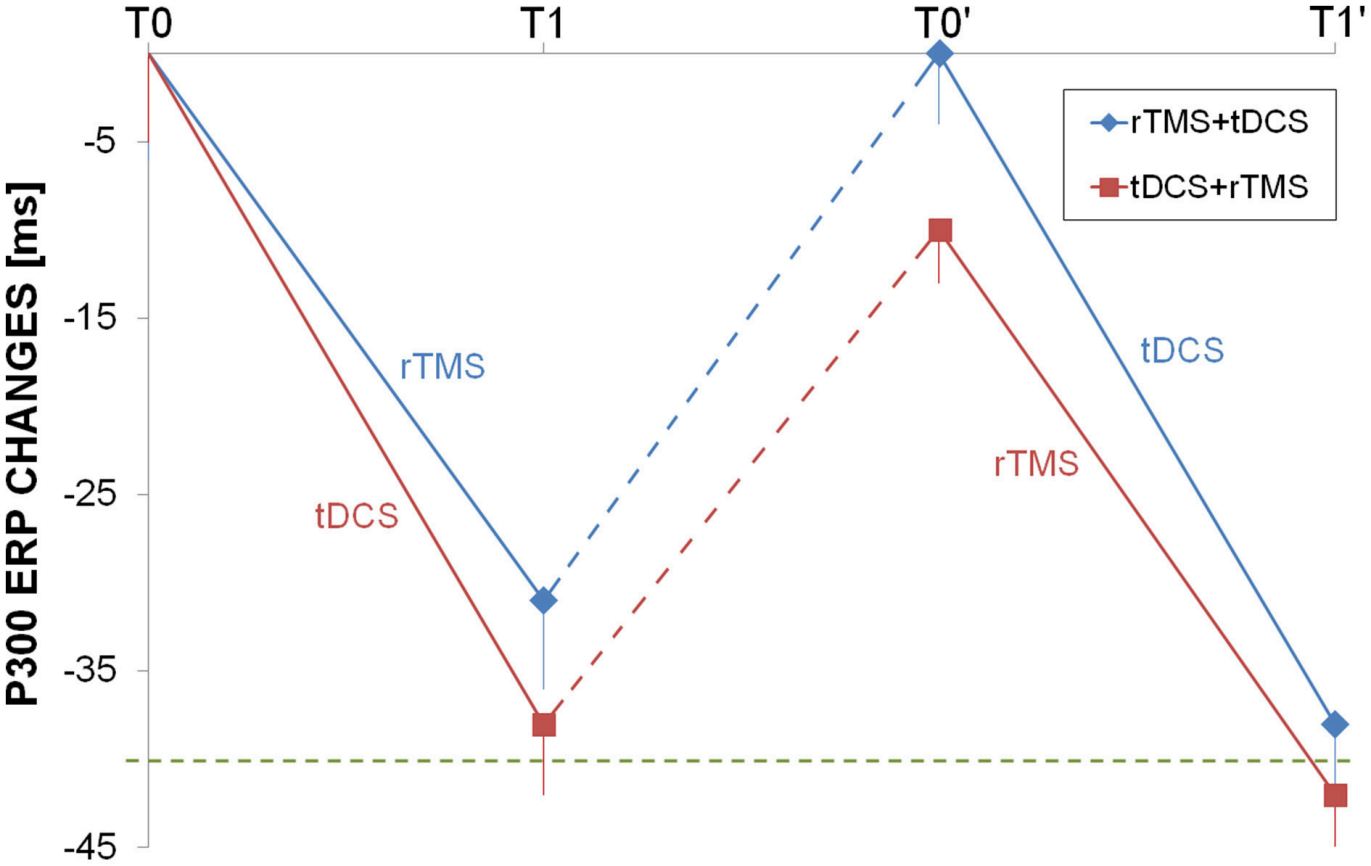
**Longitudinal auditory evoked potential evaluation**. P300 changes from baseline were shown for rTMS+tDCS (blue diamonds) and tDCS+rTMS (red squares) groups. Dotted blue and red lines indicated washout. The dotted green line was at 40 ms improvement as reference. Abbreviations as in Figure 1.

N200 and P300 had a very similar changes induced by rTMS and tDCS, as both interventions were able to shorten the ERP but only temporally, because after the washout the latencies return to the baseline values (Figure 4).

Time had a significant effect only for four tests: the copy of figure with immediate recall was significant for the sham-tDCS group (χ^2^ = 8.0, *p* = 0.037), for the dual-tDCS group (χ^2^ = 15.9, *p* < 0.001) and for the rTMS group (χ^2^ = 8.6, *p* = 0.015). Time was a significant factor for the other three tests only in the dual-tDCS group: copy of figure with delayed recall (χ^2^ = 25.7, *p* < 0.001), attentional matrices (χ^2^ = 6.2, *p* = 0.043), and perseverations in the Nelson's MCST test (χ^2^ = 7.1, *p* = 0.027). See Table 3 for *post-hoc* tests and average differences. Only some results survived Bonferroni correction (*p* < 0.016).

**Table 3:** **Longitudinal effects of treatments onto the neuropsychological scores**.

**Time points**	**t0–t1**		**t1–t2**		**t0–t2**	
**SHAM-tDCS**						
Copy of figure immediate recall	*p =* 0.025	0.5	*p* = 0.990	NS	*p* = 0.157	NS
**rTMS**						
Copy of figure immediate recall	*p =* 0.008	0.25	*p* = 0.380	NS	*p* = 0.260	NS
**DUAL-tDCS**						
Copy of figure delayed recall	*p =* 0.001	0.36	*p* = 0.317	NS	*p =* 0.002	0.35
Copy of figure immediate recall	*p* = 0.005	0.25	*p* = 0.317	NS	*p* < 0.001	0.4
Attentional matrices	*p* = 0.050	3.6	*p* = 0.822	NS	*p* = 0.023	3.1
Nelson MCST perseveration	*p* = 0.048	−11	*p* = 0.122	NS	*p* = 0.778	NS

*t0–t1, differences between baseline and after treatment; t1–t2, differences between treatment and follow-up; t0–t2, differences between baseline and follow-up; NS, not significant; p, probability for Wilcoxon test*.

### Study 3—cognitive differences in patients that responded to motor rehabilitation

At baseline, after one or two cycles of treatment there were no differences in ERP or NPS between responders and no-responders.

## Discussion

In partial disagreement with previous results (Patel et al., 2002; Mehta et al., 2003; Saxena et al., 2007; Rabadi et al., 2008), the present study shows improvements in motor and cognitive performances even in patients with chronic stroke presenting some cognitive deficits (responders and no-responders did not differ for ERP or NPS at baseline) after NIBS treatment.

In the present study patients with cognitive impairment (MMSE < 25) have been excluded, in order to reduce confounding factors, but focal deficits were detected in some patients, mostly with left hemisphere lesions (Tables 1, 2). This finding could be explained by many factors. First, we evaluated many different cognitive domains and did not rely only on less sensitive screening tests as in the majority of previous studies (Carter et al., 1988; Barker-Collo et al., 2009; Winkens et al., 2009; Hoffmann et al., 2010; McPhail et al., 2014). Furthermore NIBS stimulations (rTMS or dual-tDCS), producing long lasting effect on cortical plasticity (Miniussi et al., 2008), could promote motor and cognitive improvement also in chronic patients, who traditionally are believed to be stable and not suitable for rehabilitation. The impairment in some cognitive domains should, thus, not be considered an exclusion criterion for rehabilitation in NIBS training programs.

The interventions were safe and tolerated (Table 1) and had a partial efficacy.

Motor improvement in hands' functionality, measured with ARAT, was observed after NIBS treatment in a large percentage of patients (44%), but not after sham (Figure 2). The effect was stable in time (baseline < intervention and follow-up) and it was similar for dual-tDCS and rTMS at every time point (Figure 3). Looking at the additive effects of two cycles of intervention we can observe that, regardless the techniques used first for priming, the conjoint effects were significant on the clinical outcome (ARAT difference > MCID/MCD 3.5–5.7).

ERP endogenous components (N200 and P300) reflect perceptual and cognitive processing and can play an important role in testing stroke patients (Hillyard and Kutas, 1983); for instance, Alonso-Prieto et al. (2002) demonstrated the high sensitivity of P300 in detecting alterations of sustained attention in stroke patients with right parietal lesion.

Stahlhut et al. (2014) reported ERP data of 563 stroke patients within 4 weeks, after 12 and 24 months from the ischemic event. In this paper, a lengthened P300 latency at baseline in 51% of the patients, similar for left or right lesions, was reported with a significant improvement after 24 months (about 20 ms), similar for left and right hemispheric infarction.

In previous reports, the authors measured P300 latencies at Pz or Cz (Trinka et al., 2000; Alonso-Prieto et al., 2002; Yamagata et al., 2004; Stahlhut et al., 2014) while in our study we used a finer setup and ERP latencies has been computed at F3, F4, F7, F8, C3, C4, P3, P4, T3, T4. No significant difference has been observed in P300 latency values measured on right or left hemisphere's electrodes. The finding of P300 latency lengthening in the baseline EEG mirrored the focal impairment in attention domain that has been observed in many of our patients (Table 2). rTMS or dual-tDCS + MT, compared to sham-tDCS, had similar effects: treated patients showed a significant improvement in P300 latencies (of about 30 ms), but the improvement was transient and lost after the 6-months washout period.

The positive effect on P300 latency, after NIBS, is an additional unspecific improvement, linked to attentional networks functionality, achieved by transient modification of neuronal plasticity. Although not permanent, it could be used in the rehabilitation of chronic patients because it could produce a greater compliance and it should be possibly used to promote simultaneous neurocognitive training (e.g., visuospatial skills and MT). On the other hand, ERP were not useful to predict long term outcome and to identify responders or as a surrogate quantitative marker of the effect of NIBS.

The cognitive improvement was prevalently observed in dual-tDCS + MT for tests that are mainly influenced by the visuospatial domain (spatial attention, spatial memory, visuoconstructive skills). While some results were also found in TMS or in sham + MT they were not stable at follow-up as were instead in dual-tDCS + MT. This could be interpreted as an effect of neural plasticity that strengthened and stabilized the MT rehabilitation training. This result is not surprising as MT could enhance spatial coupling, as previously argued by Michielsen et al. Indeed, they hypothesized that the mirror illusion could increase the tendency of one limb to take on the spatial properties of the other (Michielsen et al., 2011). The effects anyway were moderate (3–8%), they did not impact dramatically on clinical outcome in a single run. We could not look at conjoint effects of the two interventions as only dual-tDCS impacted on cognition in the long run.

In our study stimulation has been performed on M1 of the unaffected hemisphere with inhibitory low frequency rTMS, or on M1 of both hemispheres with dual-TMS (excitatory anodal-tDCS on affected hemisphere and inhibitory cathodal-tDCS on unaffected hemisphere). Consequently, cortical stimulation targets have been chosen in order to improve plasticity of cortical and sub-cortical motor networks and not specifically of cognitive networks. Nevertheless, a transient cognitive improvement has been observed after each NIBS technique. These observations demonstrate that the actual knowledge of physiological mechanisms underlying NIBS techniques is still very limited. In fact, many different cortical targets have been stimulated with many different NIBS techniques (single-pulse TMS, low frequency rTMS, high frequency rTMS, anodal-tDCS, cathodal-tDCS, dual-tDCS) to improve cortical plasticity in cognitive rehabilitation protocols on attention domain, but results are contradictory (Seyal et al., 1995; Oliveri et al., 1999, 2000, 2001; Hilgetag et al., 2001; Brighina et al., 2003; Shindo et al., 2006; Ragert et al., 2008). Hence, we could hypothesize that brain stimulation effects on neuronal activity of a specific target areas are wide and not easily predictable. Moreover, even if physiological mechanisms of rTMS and tDCS are known to be different (Schlaug et al., 2008; Lefaucheur et al., 2014; Lüdemann-Podubecká et al., 2014), we did not detected any difference in motor or ERP improvement. Only one previous work (Simis et al., 2013) led a comparison between these two NIBS techniques in healthy subjects, observing that both techniques induced similar motor gains, but opposite results in cortical excitability, confirming the lack of complete understanding of the physiological processes induced by NIBS. In our study the observed effects of NIBS may be related to the direct change of activity in brain areas immediately beneath the stimulation site or, more probably, may involve more extensively connected neural networks. This is supported by previous works that demonstrated that rTMS and anodal-tDCS can induce modification of cortical activity both locally and in distant sites (Lang et al., 2005; Sack et al., 2007; Ruff et al., 2008).

Even if the precise mechanisms underlying NIBS techniques remain unclear, the modification of cortical excitability may promote adaptive neural reorganization or interrupt maladaptive functional mechanisms. These mechanisms, such as inter-hemispheric inhibition, can limit recovery by inhibition of perilesional brain areas (Murase et al., 2004; Ward and Cohen, 2004), restoring a correct balance between damaged and undamaged hemisphere and promoting behavioral recovery (Pascual-Leone, 2006; Miniussi et al., 2008). Furthermore, even if this facilitatory effect is transient, NIBS application in concomitance with rehabilitative training that supports learning processes may perpetuate the behavioral effects further, beyond the end of stimulation (Rossi and Rossini, 2004; Pascual-Leone, 2006).

The observation that rTMS and dual-tDCS have similar effects on brain plasticity could have important practical implications in neuro-rehabilitation. First, tDCS has some advantages, it is a simple and portable device, it is a non-expensive procedure, painless, and without severe collateral effects. Moreover, tDCS devices are wearable and can be used to stimulate patients online during more complex motor or cognitive training also in parallel, as in our experiment. Finally, tDCS allows an easy and reliable sham condition, which allows double blind clinical trials. However, its major limitation is that it is not as focal as TMS, so it does not allow an accurate mapping of cortical areas. Nevertheless, our data showed that it is possible to obtain satisfactory results integrating tDCS with the MT directing the modulatory effects onto the upper limb and, at the same time, improving cognitive performances.

This study suggests that the slow improvement in motor learning due to the memory stabilization (Wessel et al., 2015) could be an important factor in NIBS rehabilitation, in addition to other parameters. Also the number of cycles and the interval between them should be considered and investigated in future. The great variability in the response to NIBS, shown even by healthy subjects (Wiethoff et al., 2014; Strube et al., 2015), compels investigators to find reliable predictors of induced plasticity changes (e.g., neuroimaging characteristics, clinical features) in patients undergoing rehabilitation.

## Study limitations

Some limits should be taken in considerations:

Our sample was relatively small and heterogeneous, so it should be replied in a larger randomized clinical trial. The best candidate is a MT intervention with real and sham dual-tDCS.
The study protocol did not include imaging (CT or MRI), so it was impossible to provide a precise functional map of the damaged cortical and subcortical areas.
There were some differences in N200 and P300 at baseline in the different groups.
rTMS was underpowered compared to tDCS as in the second arm two interventions (tDCS + MT) were administered, but it was not feasible to use MT online with TMS;
The poor knowledge of physiologic mechanisms could limit the interpretations of our results.

## Conclusions

The present study allows some practical considerations, useful for neuro-rehabilitation:

First the impairment in some cognitive domains cannot be considered an exclusion criterion for rehabilitation with NIBS.
Second, NIBS generally improved ERP, but transitorily.
Third, attentive processes depend on different cortical areas and may improve with brain stimulation, also on M1, perhaps because of restoring the hemispheric balance or by distant connections effects.
Finally, NIBS effects were comparable, but there are some advantages of using tDCS vs. rTMS in stroke rehabilitation. More than one NIBS cycle (2–4 weeks) should be used in rehabilitation to obtain clinical relevant results after a washout period only in responder patients.

## Author contributions

All authors listed, have made substantial, direct and intellectual contribution to the work, and approved it for publication.

### Conflict of interest statement

The authors declare that the research was conducted in the absence of any commercial or financial relationships that could be construed as a potential conflict of interest.
